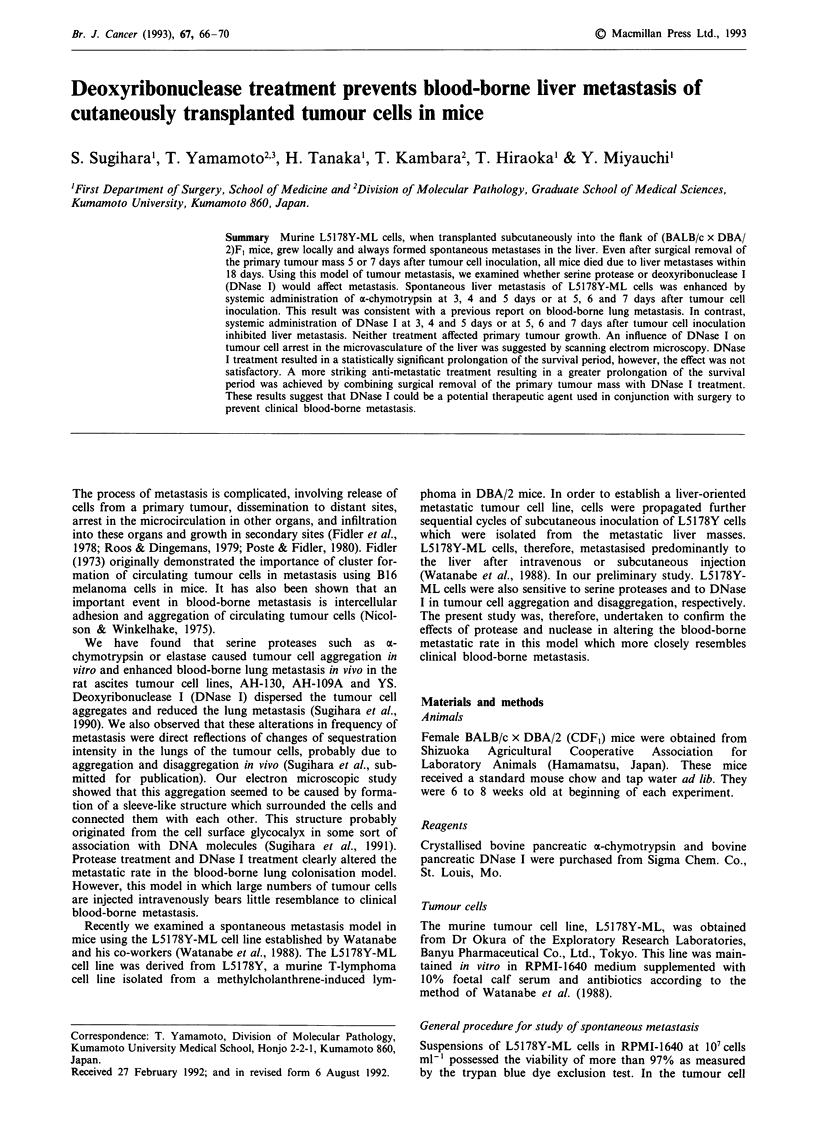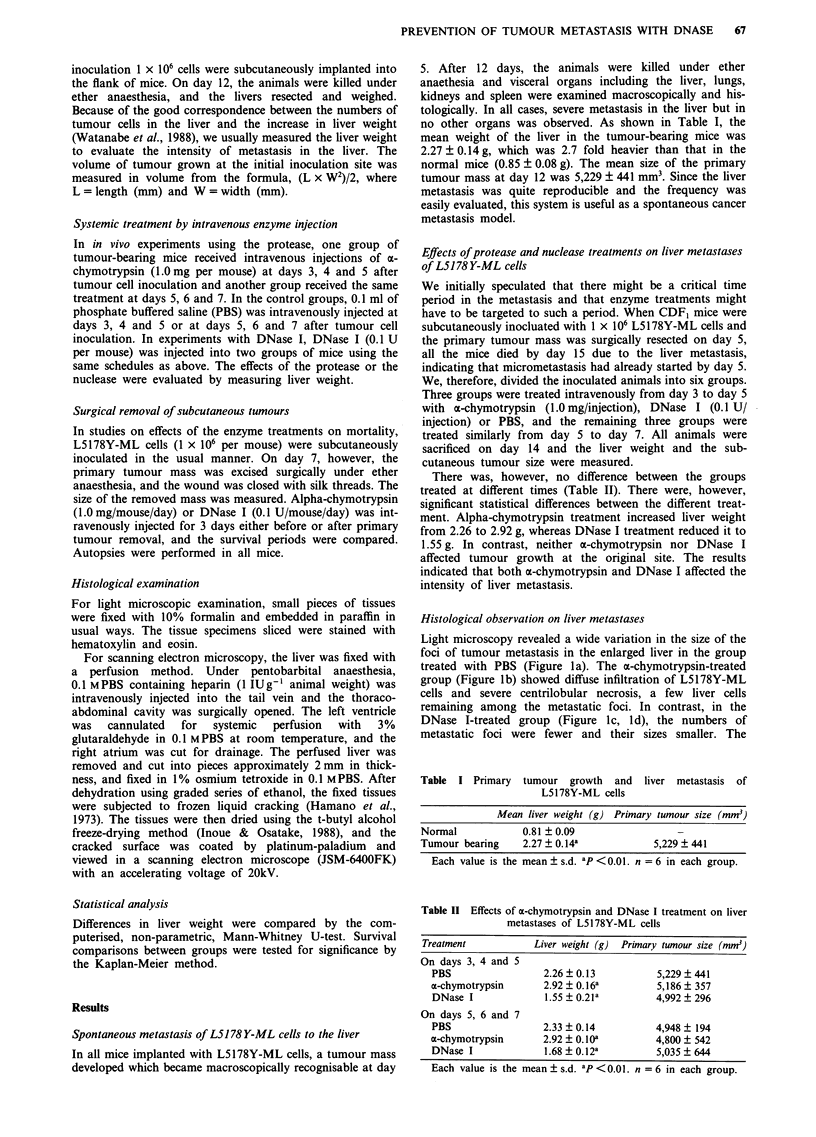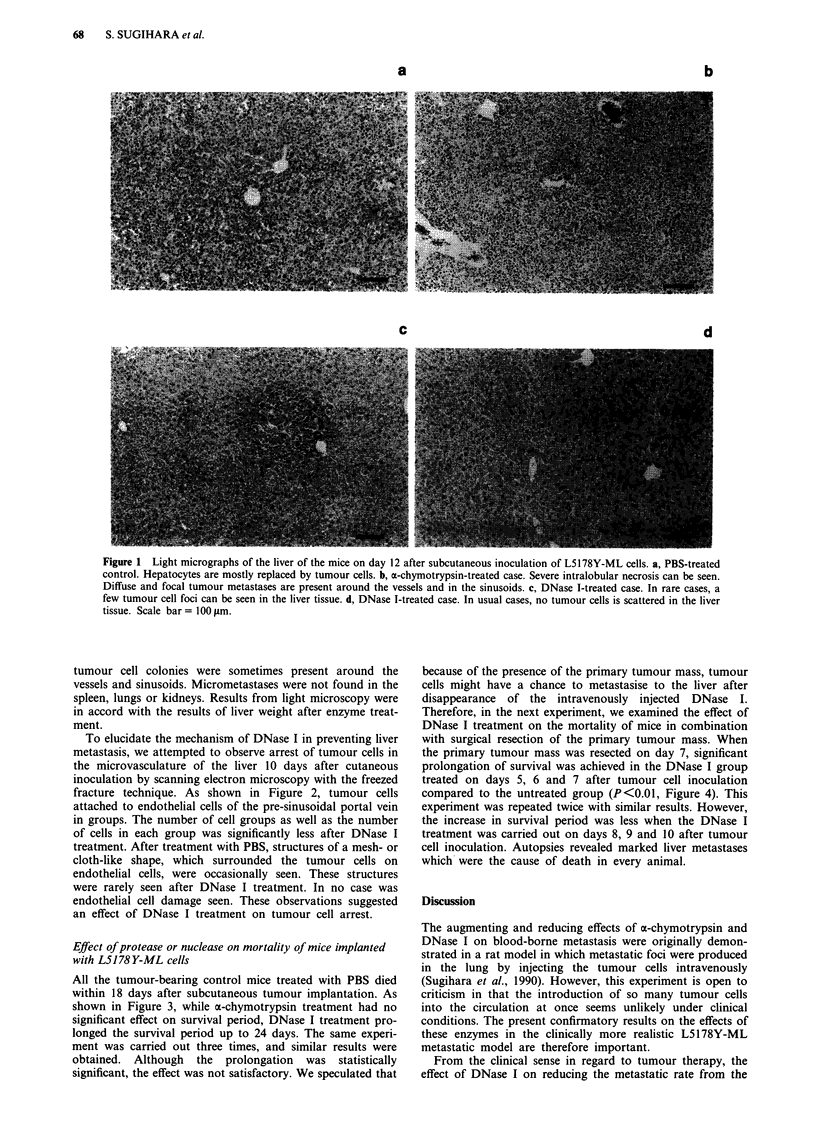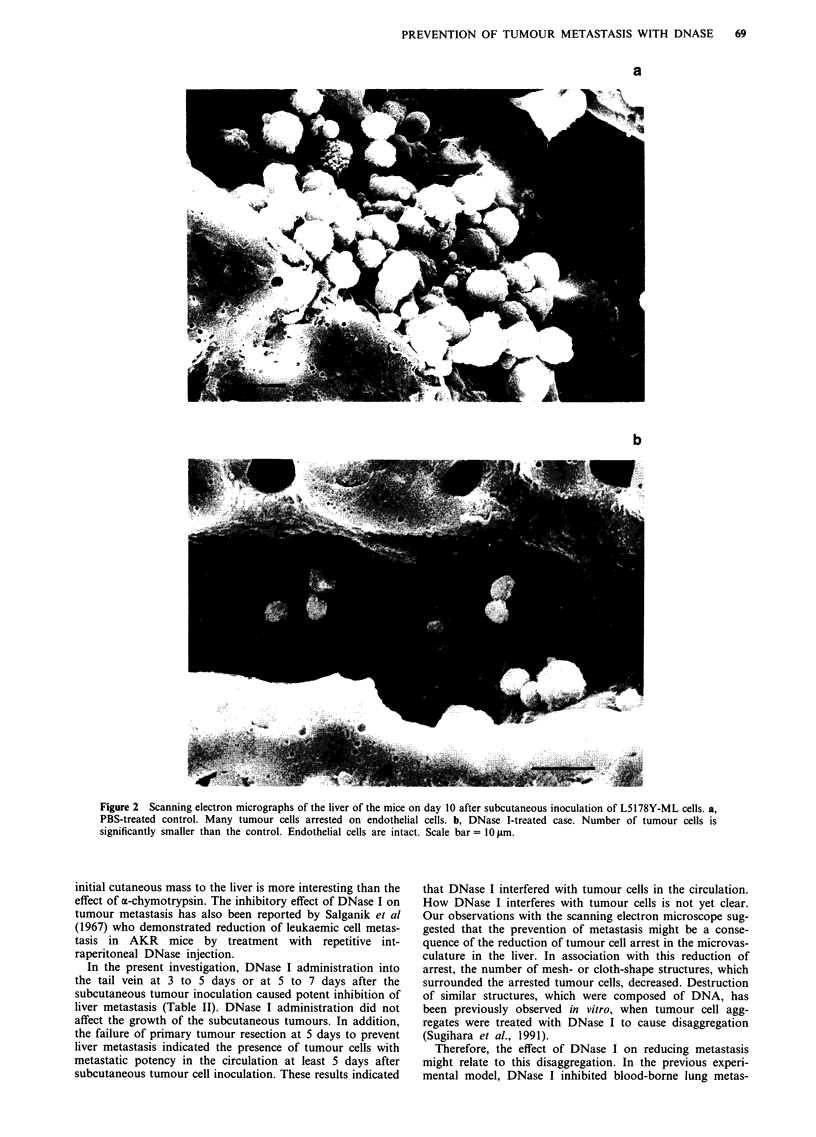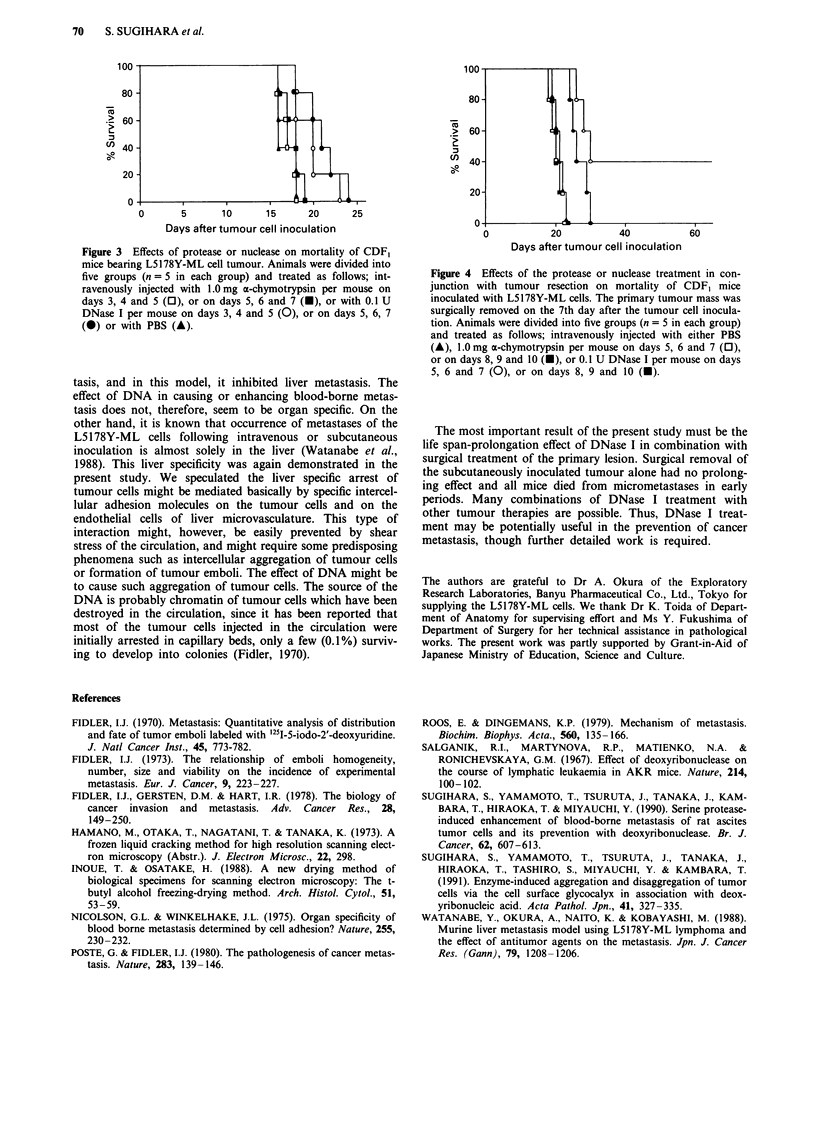# Deoxyribonuclease treatment prevents blood-borne liver metastasis of cutaneously transplanted tumour cells in mice.

**DOI:** 10.1038/bjc.1993.10

**Published:** 1993-01

**Authors:** S. Sugihara, T. Yamamoto, H. Tanaka, T. Kambara, T. Hiraoka, Y. Miyauchi

**Affiliations:** First Department of Surgery, School of Medicine, Kumamoto University, Japan.

## Abstract

**Images:**


Br. J. Cancer (1993), 67, 66-70 © Macmillan Press Ltd., 1993

### Deoxyribonuclease treatment prevents blood-borne liver metastasis of cutaneously transplanted tumour cells in mice

S. Sugihara[1], T. Yamamoto[2,3], H. Tanaka[1], T. Kambara[2], T. Hiraoka[1] & Y. Miyauchi[1]

[1]*First Department of Surgery, School of Medicine and* [2]*Division of Molecular Pathology, Graduate School of Medical Sciences, Kumamoto University, Kumamoto 860, Japan.*

**Summary** Murine L5178Y-ML cells, when transplanted subcutaneously into the flank of (BALB/c × DBA/2)$F_1$ mice, grew locally and always formed spontaneous metastases in the liver. Even after surgical removal of the primary tumour mass 5 or 7 days after tumour cell inoculation, all mice died due to liver metastases within 18 days. Using this model of tumour metastasis, we examined whether serine protease or deoxyribonuclease I (DNase I) would affect metastasis. Spontaneous liver metastasis of L5178Y-ML cells was enhanced by systemic administration of α-chymotrypsin at 3, 4 and 5 days or at 5, 6 and 7 days after tumour cell inoculation. This result was consistent with a previous report on blood-borne lung metastasis. In contrast, systemic administration of DNase I at 3, 4 and 5 days or at 5, 6 and 7 days after tumour cell inoculation inhibited liver metastasis. Neither treatment affected primary tumour growth. An influence of DNase I on tumour cell arrest in the microvasculature of the liver was suggested by scanning electrom microscopy. DNase I treatment resulted in a statistically significant prolongation of the survival period, however, the effect was not satisfactory. A more striking anti-metastatic treatment resulting in a greater prolongation of the survival period was achieved by combining surgical removal of the primary tumour mass with DNase I treatment. These results suggest that DNase I could be a potential therapeutic agent used in conjunction with surgery to prevent clinical blood-borne metastasis.

The process of metastasis is complicated, involving release of cells from a primary tumour, dissemination to distant sites, arrest in the microcirculation in other organs, and infiltration into these organs and growth in secondary sites (Fidler *et al.*, 1978; Roos & Dingemans, 1979; Poste & Fidler, 1980). Fidler (1973) originally demonstrated the importance of cluster formation of circulating tumour cells in metastasis using B16 melanoma cells in mice. It has also been shown that an important event in blood-borne metastasis is intercellular adhesion and aggregation of circulating tumour cells (Nicolson & Winkelhake, 1975).

We have found that serine proteases such as α-chymotrypsin or elastase caused tumour cell aggregation *in vitro* and enhanced blood-borne lung metastasis *in vivo* in the rat ascites tumour cell lines, AH-130, AH-109A and YS. Deoxyribonuclease I (DNase I) dispersed the tumour cell aggregates and reduced the lung metastasis (Sugihara *et al.*, 1990). We also observed that these alterations in frequency of metastasis were direct reflections of changes of sequestration intensity in the lungs of the tumour cells, probably due to aggregation and disaggregation *in vivo* (Sugihara *et al.*, submitted for publication). Our electron microscopic study showed that this aggregation seemed to be caused by formation of a sleeve-like structure which surrounded the cells and connected them with each other. This structure probably originated from the cell surface glycocalyx in some sort of association with DNA molecules (Sugihara *et al.*, 1991). Protease treatment and DNase I treatment clearly altered the metastatic rate in the blood-borne lung colonisation model. However, this model in which large numbers of tumour cells are injected intravenously bears little resemblance to clinical blood-borne metastasis.

Recently we examined a spontaneous metastasis model in mice using the L5178Y-ML cell line established by Watanabe and his co-workers (Watanabe *et al.*, 1988). The L5178Y-ML cell line was derived from L5178Y, a murine T-lymphoma cell line isolated from a methylcholanthrene-induced lymphoma in DBA/2 mice. In order to establish a liver-oriented metastatic tumour cell line, cells were propagated further sequential cycles of subcutaneous inoculation of L5178Y cells which were isolated from the metastatic liver masses. L5178Y-ML cells, therefore, metastasised predominantly to the liver after intravenous or subcutaneous injection (Watanabe *et al.*, 1988). In our preliminary study. L5178Y-ML cells were also sensitive to serine proteases and to DNase I in tumour cell aggregation and disaggregation, respectively. The present study was, therefore, undertaken to confirm the effects of protease and nuclease in altering the blood-borne metastatic rate in this model which more closely resembles clinical blood-borne metastasis.

### Materials and methods

### *Animals*

Female BALB/c × DBA/2 ($CDF_1$) mice were obtained from Shizuoka Agricultural Cooperative Association for Laboratory Animals (Hamamatsu, Japan). These mice received a standard mouse chow and tap water *ad lib.* They were 6 to 8 weeks old at beginning of each experiment.

### *Reagents*

Crystallised bovine pancreatic α-chymotrypsin and bovine pancreatic DNase I were purchased from Sigma Chem. Co., St. Louis, Mo.

### *Tumour cells*

The murine tumour cell line, L5178Y-ML, was obtained from Dr Okura of the Exploratory Research Laboratories, Banyu Pharmaceutical Co., Ltd., Tokyo. This line was maintained *in vitro* in RPMI-1640 medium supplemented with 10% foetal calf serum and antibiotics according to the method of Watanabe *et al.* (1988).

### *General procedure for study of spontaneous metastasis*

Suspensions of L5178Y-ML cells in RPMI-1640 at $10^7$ cells $ml^{-1}$ possessed the viability of more than 97% as measured by the trypan blue dye exclusion test. In the tumour cell

Correspondence: T. Yamamoto, Division of Molecular Pathology, Kumamoto University Medical School, Honjo 2-2-1, Kumamoto 860, Japan.
Received 27 February 1992; and in revised form 6 August 1992.

inoculation $1 \times 10^6$ cells were subcutaneously implanted into the flank of mice. On day 12, the animals were killed under ether anaesthesia, and the livers resected and weighed. Because of the good correspondence between the numbers of tumour cells in the liver and the increase in liver weight (Watanabe *et al.*, 1988), we usually measured the liver weight to evaluate the intensity of metastasis in the liver. The volume of tumour grown at the initial inoculation site was measured in volume from the formula, $(L \times W^2)/2$, where L = length (mm) and W = width (mm).

*Systemic treatment by intravenous enzyme injection*

In *in vivo* experiments using the protease, one group of tumour-bearing mice received intravenous injections of α-chymotrypsin (1.0 mg per mouse) at days 3, 4 and 5 after tumour cell inoculation and another group received the same treatment at days 5, 6 and 7. In the control groups, 0.1 ml of phosphate buffered saline (PBS) was intravenously injected at days 3, 4 and 5 or at days 5, 6 and 7 after tumour cell inoculation. In experiments with DNase I, DNase I (0.1 U per mouse) was injected into two groups of mice using the same schedules as above. The effects of the protease or the nuclease were evaluated by measuring liver weight.

*Surgical removal of subcutaneous tumours*

In studies on effects of the enzyme treatments on mortality, L5178Y-ML cells ($1 \times 10^6$ per mouse) were subcutaneously inoculated in the usual manner. On day 7, however, the primary tumour mass was excised surgically under ether anaesthesia, and the wound was closed with silk threads. The size of the removed mass was measured. Alpha-chymotrypsin (1.0 mg/mouse/day) or DNase I (0.1 U/mouse/day) was intravenously injected for 3 days either before or after primary tumour removal, and the survival periods were compared. Autopsies were performed in all mice.

*Histological examination*

For light microscopic examination, small pieces of tissues were fixed with 10% formalin and embedded in paraffin in usual ways. The tissue specimens sliced were stained with hematoxylin and eosin.

For scanning electron microscopy, the liver was fixed with a perfusion method. Under pentobarbital anaesthesia, 0.1 M PBS containing heparin ($1\ IU\,g^{-1}$ animal weight) was intravenously injected into the tail vein and the thoraco-abdominal cavity was surgically opened. The left ventricle was cannulated for systemic perfusion with 3% glutaraldehyde in 0.1 M PBS at room temperature, and the right atrium was cut for drainage. The perfused liver was removed and cut into pieces approximately 2 mm in thickness, and fixed in 1% osmium tetroxide in 0.1 M PBS. After dehydration using graded series of ethanol, the fixed tissues were subjected to frozen liquid cracking (Hamano *et al.*, 1973). The tissues were then dried using the t-butyl alcohol freeze-drying method (Inoue & Osatake, 1988), and the cracked surface was coated by platinum-paladium and viewed in a scanning electron microscope (JSM-6400FK) with an accelerating voltage of 20kV.

*Statistical analysis*

Differences in liver weight were compared by the computerised, non-parametric, Mann-Whitney U-test. Survival comparisons between groups were tested for significance by the Kaplan-Meier method.

### Results

*Spontaneous metastasis of L5178Y-ML cells to the liver*

In all mice implanted with L5178Y-ML cells, a tumour mass developed which became macroscopically recognisable at day 5. After 12 days, the animals were killed under ether anaethesia and visceral organs including the liver, lungs, kidneys and spleen were examined macroscopically and histologically. In all cases, severe metastasis in the liver but in no other organs was observed. As shown in Table I, the mean weight of the liver in the tumour-bearing mice was 2.27 ± 0.14 g, which was 2.7 fold heavier than that in the normal mice (0.85 ± 0.08 g). The mean size of the primary tumour mass at day 12 was 5,229 ± 441 $mm^3$. Since the liver metastasis was quite reproducible and the frequency was easily evaluated, this system is useful as a spontaneous cancer metastasis model.

*Effects of protease and nuclease treatments on liver metastases of L5178Y-ML cells*

We initially speculated that there might be a critical time period in the metastasis and that enzyme treatments might have to be targeted to such a period. When $CDF_1$ mice were subcutaneously inocluated with $1 \times 10^6$ L5178Y-ML cells and the primary tumour mass was surgically resected on day 5, all the mice died by day 15 due to the liver metastasis, indicating that micrometastasis had already started by day 5. We, therefore, divided the inoculated animals into six groups. Three groups were treated intravenously from day 3 to day 5 with α-chymotrypsin (1.0 mg/injection), DNase I (0.1 U/injection) or PBS, and the remaining three groups were treated similarly from day 5 to day 7. All animals were sacrificed on day 14 and the liver weight and the subcutaneous tumour size were measured.

There was, however, no difference between the groups treated at different times (Table II). There were, however, significant statistical differences between the different treatment. Alpha-chymotrypsin treatment increased liver weight from 2.26 to 2.92 g, whereas DNase I treatment reduced it to 1.55 g. In contrast, neither α-chymotrypsin nor DNase I affected tumour growth at the original site. The results indicated that both α-chymotrypsin and DNase I affected the intensity of liver metastasis.

*Histological observation on liver metastases*

Light microscopy revealed a wide variation in the size of the foci of tumour metastasis in the enlarged liver in the group treated with PBS (Figure 1a). The α-chymotrypsin-treated group (Figure 1b) showed diffuse infiltration of L5178Y-ML cells and severe centrilobular necrosis, a few liver cells remaining among the metastatic foci. In contrast, in the DNase I-treated group (Figure 1c, 1d), the numbers of metastatic foci were fewer and their sizes smaller. The

**Table I** Primary tumour growth and liver metastasis of L5178Y-ML cells

| | *Mean liver weight (g)* | *Primary tumour size ($mm^3$)* |
|---|---|---|
| Normal | 0.81 ± 0.09 | – |
| Tumour bearing | 2.27 ± 0.14[a] | 5,229 ± 441 |

Each value is the mean ± s.d. [a]$P < 0.01$. $n = 6$ in each group.

**Table II** Effects of α-chymotrypsin and DNase I treatment on liver metastases of L5178Y-ML cells

| *Treatment* | *Liver weight (g)* | *Primary tumour size ($mm^3$)* |
|---|---|---|
| On days 3, 4 and 5 | | |
| PBS | 2.26 ± 0.13 | 5,229 ± 441 |
| α-chymotrypsin | 2.92 ± 0.16[a] | 5,186 ± 357 |
| DNase I | 1.55 ± 0.21[a] | 4,992 ± 296 |
| On days 5, 6 and 7 | | |
| PBS | 2.33 ± 0.14 | 4,948 ± 194 |
| α-chymotrypsin | 2.92 ± 0.10[a] | 4,800 ± 542 |
| DNase I | 1.68 ± 0.12[a] | 5,035 ± 644 |

Each value is the mean ± s.d. [a]$P < 0.01$. $n = 6$ in each group.

**Figure 1** Light micrographs of the liver of the mice on day 12 after subcutaneous inoculation of L5178Y-ML cells. **a**, PBS-treated control. Hepatocytes are mostly replaced by tumour cells. **b**, α-chymotrypsin-treated case. Severe intralobular necrosis can be seen. Diffuse and focal tumour metastases are present around the vessels and in the sinusoids. **c**, DNase I-treated case. In rare cases, a few tumour cell foci can be seen in the liver tissue. **d**, DNase I-treated case. In usual cases, no tumour cells is scattered in the liver tissue. Scale bar = 100 μm.

tumour cell colonies were sometimes present around the vessels and sinusoids. Micrometastases were not found in the spleen, lungs or kidneys. Results from light microscopy were in accord with the results of liver weight after enzyme treatment.

To elucidate the mechanism of DNase I in preventing liver metastasis, we attempted to observe arrest of tumour cells in the microvasculature of the liver 10 days after cutaneous inoculation by scanning electron microscopy with the freezed fracture technique. As shown in Figure 2, tumour cells attached to endothelial cells of the pre-sinusoidal portal vein in groups. The number of cell groups as well as the number of cells in each group was significantly less after DNase I treatment. After treatment with PBS, structures of a mesh- or cloth-like shape, which surrounded the tumour cells on endothelial cells, were occasionally seen. These structures were rarely seen after DNase I treatment. In no case was endothelial cell damage seen. These observations suggested an effect of DNase I treatment on tumour cell arrest.

*Effect of protease or nuclease on mortality of mice implanted with L5178Y-ML cells*

All the tumour-bearing control mice treated with PBS died within 18 days after subcutaneous tumour implantation. As shown in Figure 3, while α-chymotrypsin treatment had no significant effect on survival period, DNase I treatment prolonged the survival period up to 24 days. The same experiment was carried out three times, and similar results were obtained. Although the prolongation was statistically significant, the effect was not satisfactory. We speculated that because of the presence of the primary tumour mass, tumour cells might have a chance to metastasise to the liver after disappearance of the intravenously injected DNase I. Therefore, in the next experiment, we examined the effect of DNase I treatment on the mortality of mice in combination with surgical resection of the primary tumour mass. When the primary tumour mass was resected on day 7, significant prolongation of survival was achieved in the DNase I group treated on days 5, 6 and 7 after tumour cell inoculation compared to the untreated group ($P<0.01$, Figure 4). This experiment was repeated twice with similar results. However, the increase in survival period was less when the DNase I treatment was carried out on days 8, 9 and 10 after tumour cell inoculation. Autopsies revealed marked liver metastases which were the cause of death in every animal.

### Discussion

The augmenting and reducing effects of α-chymotrypsin and DNase I on blood-borne metastasis were originally demonstrated in a rat model in which metastatic foci were produced in the lung by injecting the tumour cells intravenously (Sugihara *et al.*, 1990). However, this experiment is open to criticism in that the introduction of so many tumour cells into the circulation at once seems unlikely under clinical conditions. The present confirmatory results on the effects of these enzymes in the clinically more realistic L5178Y-ML metastatic model are therefore important.

From the clinical sense in regard to tumour therapy, the effect of DNase I on reducing the metastatic rate from the

**Figure 2** Scanning electron micrographs of the liver of the mice on day 10 after subcutaneous inoculation of L5178Y-ML cells. **a**, PBS-treated control. Many tumour cells arrested on endothelial cells. **b**, DNase I-treated case. Number of tumour cells is significantly smaller than the control. Endothelial cells are intact. Scale bar = 10 $\mu$m.

initial cutaneous mass to the liver is more interesting than the effect of $\alpha$-chymotrypsin. The inhibitory effect of DNase I on tumour metastasis has also been reported by Salganik *et al* (1967) who demonstrated reduction of leukaemic cell metastasis in AKR mice by treatment with repetitive intraperitoneal DNase injection.

In the present investigation, DNase I administration into the tail vein at 3 to 5 days or at 5 to 7 days after the subcutaneous tumour inoculation caused potent inhibition of liver metastasis (Table II). DNase I administration did not affect the growth of the subcutaneous tumours. In addition, the failure of primary tumour resection at 5 days to prevent liver metastasis indicated the presence of tumour cells with metastatic potency in the circulation at least 5 days after subcutaneous tumour cell inoculation. These results indicated that DNase I interfered with tumour cells in the circulation. How DNase I interferes with tumour cells is not yet clear. Our observations with the scanning electron microscope suggested that the prevention of metastasis might be a consequence of the reduction of tumour cell arrest in the microvasculature in the liver. In association with this reduction of arrest, the number of mesh- or cloth-shape structures, which surrounded the arrested tumour cells, decreased. Destruction of similar structures, which were composed of DNA, has been previously observed *in vitro*, when tumour cell aggregates were treated with DNase I to cause disaggregation (Sugihara *et al.*, 1991).

Therefore, the effect of DNase I on reducing metastasis might relate to this disaggregation. In the previous experimental model, DNase I inhibited blood-borne lung metas-

**Figure 3** Effects of protease or nuclease on mortality of $CDF_1$ mice bearing L5178Y-ML cell tumour. Animals were divided into five groups ($n = 5$ in each group) and treated as follows; intravenously injected with 1.0 mg α-chymotrypsin per mouse on days 3, 4 and 5 (□), or on days 5, 6 and 7 (■), or with 0.1 U DNase I per mouse on days 3, 4 and 5 (○), or on days 5, 6, 7 (●) or with PBS (▲).

**Figure 4** Effects of the protease or nuclease treatment in conjunction with tumour resection on mortality of $CDF_1$ mice inoculated with L5178Y-ML cells. The primary tumour mass was surgically removed on the 7th day after the tumour cell inoculation. Animals were divided into five groups ($n = 5$ in each group) and treated as follows; intravenously injected with either PBS (▲), 1.0 mg α-chymotrypsin per mouse on days 5, 6 and 7 (□), or on days 8, 9 and 10 (■), or 0.1 U DNase I per mouse on days 5, 6 and 7 (○), or on days 8, 9 and 10 (■).

tasis, and in this model, it inhibited liver metastasis. The effect of DNA in causing or enhancing blood-borne metastasis does not, therefore, seem to be organ specific. On the other hand, it is known that occurrence of metastases of the L5178Y-ML cells following intravenous or subcutaneous inoculation is almost solely in the liver (Watanabe *et al.*, 1988). This liver specificity was again demonstrated in the present study. We speculated the liver specific arrest of tumour cells might be mediated basically by specific intercellular adhesion molecules on the tumour cells and on the endothelial cells of liver microvasculature. This type of interaction might, however, be easily prevented by shear stress of the circulation, and might require some predisposing phenomena such as intercellular aggregation of tumour cells or formation of tumour emboli. The effect of DNA might be to cause such aggregation of tumour cells. The source of the DNA is probably chromatin of tumour cells which have been destroyed in the circulation, since it has been reported that most of the tumour cells injected in the circulation were initially arrested in capillary beds, only a few (0.1%) surviving to develop into colonies (Fidler, 1970).

The most important result of the present study must be the life span-prolongation effect of DNase I in combination with surgical treatment of the primary lesion. Surgical removal of the subcutaneously inoculated tumour alone had no prolonging effect and all mice died from micrometastases in early periods. Many combinations of DNase I treatment with other tumour therapies are possible. Thus, DNase I treatment may be potentially useful in the prevention of cancer metastasis, though further detailed work is required.

The authors are grateful to Dr A. Okura of the Exploratory Research Laboratories, Banyu Pharmaceutical Co., Ltd., Tokyo for supplying the L5178Y-ML cells. We thank Dr K. Toida of Department of Anatomy for supervising effort and Ms Y. Fukushima of Department of Surgery for her technical assistance in pathological works. The present work was partly supported by Grant-in-Aid of Japanese Ministry of Education, Science and Culture.

### References

FIDLER, I.J. (1970). Metastasis: Quantitative analysis of distribution and fate of tumor emboli labeled with $^{125}$I-5-iodo-2′-deoxyuridine. *J. Natl Cancer Inst.*, **45**, 773-782.

FIDLER, I.J. (1973). The relationship of emboli homogeneity, number, size and viability on the incidence of experimental metastasis. *Eur. J. Cancer*, **9**, 223–227.

FIDLER, I.J., GERSTEN, D.M. & HART, I.R. (1978). The biology of cancer invasion and metastasis. *Adv. Cancer Res.*, **28**, 149–250.

HAMANO, M., OTAKA, T., NAGATANI, T. & TANAKA, K. (1973). A frozen liquid cracking method for high resolution scanning electron microscopy (Abstr.). *J. Electron Microsc.*, **22**, 298.

INOUE, T. & OSATAKE, H. (1988). A new drying method of biological specimens for scanning electron microscopy: The t-butyl alcohol freezing-drying method. *Arch. Histol. Cytol.*, **51**, 53–59.

NICOLSON, G.L. & WINKELHAKE, J.L. (1975). Organ specificity of blood borne metastasis determined by cell adhesion? *Nature*, **255**, 230–232.

POSTE, G. & FIDLER, I.J. (1980). The pathologenesis of cancer metastasis. *Nature*, **283**, 139–146.

ROOS, E. & DINGEMANS, K.P. (1979). Mechanism of metastasis. *Biochim. Biophys. Acta.*, **560**, 135–166.

SALGANIK, R.I., MARTYNOVA, R.P., MATIENKO, N.A. & RONICHEVSKAYA, G.M. (1967). Effect of deoxyribonuclease on the course of lymphatic leukaemia in AKR mice. *Nature*, **214**, 100–102.

SUGIHARA, S., YAMAMOTO, T., TSURUTA, J., TANAKA, J., KAMBARA, T., HIRAOKA, T. & MIYAUCHI, Y. (1990). Serine protease-induced enhancement of blood-borne metastasis of rat ascites tumor cells and its prevention with deoxyribonuclease. *Br. J. Cancer*, **62**, 607–613.

SUGIHARA, S., YAMAMOTO, T., TSURUTA, J., TANAKA, J., HIRAOKA, T., TASHIRO, S., MIYAUCHI, Y. & KAMBARA, T. (1991). Enzyme-induced aggregation and disaggregation of tumor cells via the cell surface glycocalyx in association with deoxyribonucleic acid. *Acta Pathol. Jpn.*, **41**, 327–335.

WATANABE, Y., OKURA, A., NAITO, K. & KOBAYASHI, M. (1988). Murine liver metastasis model using L5178Y-ML lymphoma and the effect of antitumor agents on the metastasis. *Jpn. J. Cancer Res. (Gann)*, **79**, 1208–1206.